# Induction of Interferon-Stimulated Genes on the IL-4 Response Axis by Epstein-Barr Virus Infected Human B Cells; Relevance to Cellular Transformation

**DOI:** 10.1371/journal.pone.0064868

**Published:** 2013-05-27

**Authors:** Nikki Smith, Rosemary Tierney, Wenbin Wei, Martina Vockerodt, Paul G. Murray, Ciaran B. Woodman, Martin Rowe

**Affiliations:** School of Cancer Sciences, Birmingham Cancer Research UK Centre, and Birmingham Centre for Human Virology, University of Birmingham College of Medical and Dental Sciences, Birmingham, United Kingdom; United Arab Emirates University, United Arab Emirates

## Abstract

Epstein-Barr virus (EBV) is an oncogenic virus that is associated with the pathogenesis of several human lymphoid malignancies, including Hodgkin's lymphoma. Infection of normal resting B cells with EBV results in activation to lymphoblasts that are phenotypically similar to those generated by physiological stimulation with CD40L plus IL-4. One important difference is that infection leads to the establishment of permanently growing lymphoblastoid cell lines, whereas CD40L/IL-4 blasts have finite proliferation lifespans. To identify early events which might later determine why EBV infected blasts go on to establish transformed cell lines, we performed global transcriptome analyses on resting B cells and on EBV and CD40L/IL-4 blasts after 7 days culture. As anticipated there was considerable overlap in the transcriptomes of the two types of lymphoblasts when compared to the original resting B cells, reflecting common changes associated with lymphocyte activation and proliferation. Of interest to us was a subset of 255 genes that were differentially expressed between EBV and CD40L/IL-4 blasts. Genes which were more highly expressed in EBV blasts were substantially and significantly enriched for a set of interferon-stimulated genes which on further *in silico* analyses were found to be repressed by IL-4 in other cell contexts and to be up-regulated in micro-dissected malignant cells from Hodgkin's lymphoma biopsies when compared to their normal germinal center cell counterparts. We hypothesized that EBV and IL-4 were targeting and discordantly regulating a common set of genes. This was supported experimentally in our B cell model where IL-4 stimulation partially reversed transcriptional changes which follow EBV infection and it impaired the efficiency of EBV-induced B cell transformation. Taken together, these data suggest that the discordant regulation of interferon and IL-4 pathway genes by EBV that occurs early following infection of B cells has relevance to the development or maintenance of an EBV-associated malignancy.

## Introduction

The normal process of B lymphocyte activation in response to encountering antigen involves complex cellular and cytokine interactions in the germinal centers of peripheral lymphoid tissues, leading to clonal expansion followed by differentiation into effector or memory cells. Some aspects of this self -limiting proliferation can be replicated *in vitro* through combinations of signals; one widely-used method of generating B cell blast involves co-stimulation with CD40L and IL-4. In this context, the pleiotropic IL-4 cytokine acts as an essential proliferation signal [1], [2], although its effects on malignant cells can be anti-proliferative and pro-apoptotic [3]–[10].

In contrast to CD40L/IL4 B blasts, which usually have finite proliferative lifespans [11], experimental infection of resting B cells with Epstein-Barr virus (EBV) in culture regularly results in the establishment of lymphoblastoid cell lines (LCL) with indefinite proliferation potential [12], [13]. EBV is therefore a potent transforming agent, and it is unsurprising that the virus is aetiologically associated with lymphoproliferative diseases, including the B cell tumors: Burkitt's lymphoma, Hodgkin's lymphoma (HL), diffuse large B cell lymphoma of the elderly, and post-transplant lymphoproliferative disease [14].

EBV is a herpesvirus with a DNA genome containing in the region of 90 genes [15]. However, only 9 or 10 viral proteins are regularly expressed in LCLs, which generally display a non-productive or ‘latent’ infection [16]. Of the transformation-associated latent viral proteins, at least one, LMP1, is oncogenic by classical criteria; its ectopic expression in model rodent fibroblast lines leads to cellular transformation *in vitro* and acquisition of a tumorigenic phenotype *in vivo* [17], [18]. Although showing no overall sequence homology with any cellular proteins, LMP1 functions as a constitutively-active member of the tumor necrosis factor receptor family which includes CD40 [19]. LMP2A is a trans-membrane signaling protein that behaves as a constitutively active functional homologue of the B cell receptor [20], [21]. The remaining transformation-associated proteins are mostly nuclear proteins (EBNA1, EBNA2, EBNA3A, EBNA3B, EBNA3C and EBNA-LP), which again show no significant homology to cellular proteins although their effects on regulation of cellular transcription and signaling pathways are well-studied [15]. Transformation of B cells by EBV cannot be attributed to a single viral oncogene. Rather, the cooperative functions of multiple viral genes are required, as demonstrated by studies with recombinant EBVs, which show that deletion of any one of EBNA1, EBNA2, EBNA3A, EBNA3C or LMP1 genes will abolish or substantially reduce transforming ability [22], [23].

Whilst the functions of the transformation-associated EBV genes have been the subject of considerable research over the last 2 or 3 decades, some fundamental questions remain unanswered. One of these concerns why EBV-infected normal B cells are transformed into blasts that grow indefinitely, whilst B cell blasts generated, for example by continuous stimulation with CD40L and IL-4, have finite proliferative potential lasting a few weeks. This is an important question as it impacts on our understanding of the role of EBV in the pathogenesis of B cell tumors.

As part of a strategy to address this question, we compared the global transcriptomes of these two types of B blasts at 7 days after virus infection or CD40L/IL-4 stimulation of normal peripheral blood B lymphocytes. Consistent with published data indicating the phenotypic similarities of EBV- and mitogen-induced B blasts [24], we found that there was considerable overlap in their transcriptomes and that mRNA changes relative to the original resting B cells largely reflected common changes associated with lymphocyte activation and cell proliferation. Of greater interest to us was the subset of genes that was differentially expressed between EBV and CD40L/IL-4 blasts. Analysis of this latter set of genes identified an intriguing discordance of EBV and IL-4 mediated transcriptional regulation that is functionally relevant to EBV-induced cellular transformation.

## Materials and Methods

### Ethics statement

Buffy coats or apheresis cones, obtained from the National Health Service Blood and Transplant service (NHSBT) in Birmingham, were used as the source of peripheral blood for this study. Healthy blood donors were recruited according to NHSBT standard procedures, which included obtaining full consent to allow use of donations for our experiments.

### Isolation of B cells and generation of B blasts

Peripheral blood mononuclear cells (PBMCs) were isolated from buffy coat samples or apheresis cones, obtained from NHSBT, by discontinuous gradient centrifugation over Lymphoprep separation medium (Robbins Scientific). CD19^+^ B cells were isolated using magnetic CD19 Dynabeads (Dynal, Invitrogen) according to the manufacturer's recommendations. Briefly, 2×10^6^ PBMCs and 10^7^ Dynabeads per ml were co-incubated at 4°C for 30 min before isolation of B cells with bound Dynabeads by exposure to a magnet. The isolated B cells were washed 5 times before removal of the beads with DETACHaBEAD® CD19 (Dynal, Invitrogen).

Virus for infection experiments was obtained from 293 cells containing the 2089 recombinant EBV genome [25] and virus titration was carried out using real-time quantitative polymerase chain-reaction (qPCR) as previously described [26]. EBV infection of isolated CD19^+^ B cells was performed by incubation of cells at 37°C for 2 h with EBV at 100 m.o.i. (multiplicity of infection) before washing away excess virus and seeding at 2×10^6^ cells/ml in fresh medium (RPMI-1640 with 10% fetal calf serum) and culturing at 37°C. Parallel cultures of mitogen-stimulated B blasts were set up in cell culture medium containing 50 ng/ml of soluble trimeric human recombinant megaCD40L (Alexis Biochemicals) and 50 ng/ml IL-4 (ProSpec-Tany).

### EBV transformation assay

EBV transformation assays were carried out as described elsewhere [26], using 96-well plates with a fixed number of 10^3^ B cells/well and a range of virus doses (0.2–100 m.o.i.). Wells were examined for transformed colonies after 6 weeks culture.

### Microarray experiments

The mirVana™PARIS™ kit (Applied Biosystems) was used to extract total RNA from resting B cells isolated from PBMCs, and from B cells stimulated with CD40L and IL-4 for 7 days or infected with EBV at a m.o.i. of 100 and harvested 7 days post-infection. RNA quality was monitored using a Bioanalyzer 2100 (Agilent, Stockport UK) then prepared for transcriptional profiling on GeneChip Human Exon 1.0 ST Arrays (Affymetrix, High Wycombe UK) according to the manufacturer's recommendations. Briefly, 100 ng of RNA was used to transcribe single stranded cDNA using oligo dT primers. A second strand was then synthesised and the double stranded cDNA purified. This double stranded cDNA was used as a template to generate fluorescently labelled cRNA by *in vitro* transcription, which was purified and fragmented before being hybridised to the microarray chip.

Scanned images of microarray chips were analysed using the Affymetrix GeneChip Command Console. Gene level analysis of the array data was performed using Affymetrix Expression Console with the default settings of “Core: RMA-Sketch”. Differentially expressed genes were identified using LIMMA [27] with a cut-off p value of 0.01. Array annotation was based on Affymetrix NetAffx annotation release 32 (June, 2011) and gene symbols were updated according to NCBI gene database downloaded on August 26, 2011. The primary data are available from GEO under series accession number GSE45829 (http://www.ncbi.nlm.nih.gov/geo/).

### DAVID gene ontology analysis

Lists of probe-sets from the GeneChip Human Exon 1.0 ST Arrays were generated according to selection criteria indicated in the results section and were submitted to DAVID gene ontology analysis (http://david.abcc.ncifcrf.gov/home.jsp) [28], [29], selecting the PANTHER_BP_ALL chart option. The probe-lists used and the complete results of these analyses are available in Data-File S1.

### Quantitative reverse-transcriptase qPCR (RT-qPCR)

Taqman® custom array cards (Applied Biosystems) were designed to allow simultaneous quantitation of 8 RNA samples per microfluidics card with 48 unique assays. For each sample 50 ng total RNA was reverse-transcribed and loaded and run according to the manufacturer's instructions in a 7900HT PCR machine with a Low Density Array upgrade (Applied Biosystems). Data was analysed using the SDS2.2.2 software (Applied Biosystems) according to manufacturer's instructions using the ddCt method [30] and exported into Microsoft Excel for further analysis.

Individual RT-qPCR assays for cellular transcripts (see Table S1) were purchased from Applied Biosystems, and used according to the manufacturer's recommendations; cDNA was obtained by reverse-transcribing 20–400 ng total RNA with random hexamer primers.

## Results

### Comparison of the transcriptomes of EBV- and mitogen-induced B blasts

**Figure 1:**
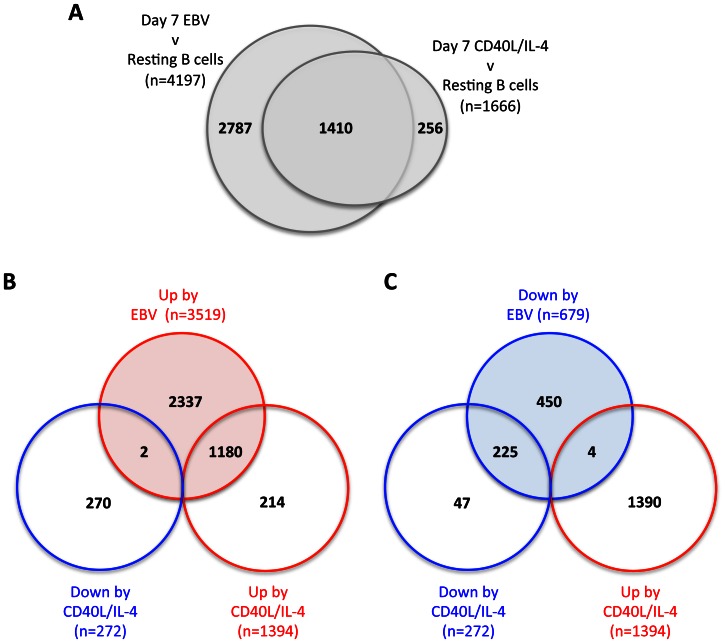
Similarities in the transcriptomes of blasts induced by EBV infection or by CD40L/IL4 treatment of resting peripheral blood B cells. Significant changes in transcript expression were identified by paired LIMMA (p<0.01; FC>1.5) and complete gene-lists are available in Data-File S2. (**A**) Venn diagram indicating the overlap of the 4197 genes changed by EBV and the 1666 genes changed by CD40L/IL4, irrespective of the direction of the change. (**B**) Venn diagram showing the 3519 genes that were up-regulated by EBV, and the overlap with the 1394 genes up-regulated by CD40L/IL4 and the 272 down-regulated by CD40L/IL4. (**C**) Venn diagram showing the 679 genes that were down-regulated by EBV, and the overlap with the 1394 genes up-regulated by CD40L/IL4 and the 272 down-regulated by CD40L/IL4.

Gene expression profiling identified 4197 and 1666 genes as significantly altered following EBV infection and CD40L/IL-4 stimulation, respectively (LIMMA; p<0.01, FC>1.5). As expected, there was a significant and substantial over-lap between these transcriptional profiles with 1410 genes altered by both EBV and mitogen (OR = 22.6, χ^2^_1df_ = 2506, p<0.0001; Figure 1A and Table 1A). Of these overlapping 1410 significant transcriptional changes, 99.6% were altered in the same direction by EBV and by CD40L/IL-4; and only 6 genes were significantly up-regulated by EBV and significantly down-regulated by CD40L/IL-4, or *vice versa* (Figures 1B, 1C and Table 1). The six discordantly regulated genes were ZNF487P and PIM2 ( up-regulated by EBV but down-regulated by CD40L/IL-4), and SPINT2, SIGLEC10, TSPAN33 and DUSP6 (down-regulated by EBV but up-regulated by CD40L/IL-4).

**Table 1:** Similarities in gene-expression changes induced by EBV and CD40L/IL4.

Comparisons between gene sets*			overlap between gene-sets	Odds Ratio	Chi-Square	P Value (1 df)
**A.**	Altered by EBV (n = 4197) and:	Altered by CD40L/IL4 (n = 1666)	1410	22.6	2506	<0.0001
**B.**	Up-regulated by EBV (n = 3519) and:	Up-regulated by CD40L/IL4 (n = 1394)	1180	29.2	2837	<0.0001
		Down-regulated by CD40L/IL4 (n = 272)	2	0.028	51.4	<0.0001
**C.**	Down-regulated by EBV (n = 679) and:	Up-regulated by CD40L/IL4 (n = 1394)	4	0.065	47.0	<0.0001
		Down-regulated by CD40L/IL4 (n = 272)	225	172	4306	<0.0001

* Numbers in parentheses indicate the number of genes within the gene-set. Note that for one gene (ZNF487P), one probe-set was up- regulated and one was down-regulated by EBV compared to resting B cells.

Genes altered by EBV or by CD40L/IL4 on day 7 relative to resting B cells at day 0 were determined by a paired LIMMA analysis with a significance threshold of P<0.01; FC>1.5. The Odds Ratio (OR) and significance of the overlaps were determined using the denominator, 17311, representing the total number of unique annotated genes on the HuExon 1.0 ST array. Complete gene-lists are available in Data-File S2.

So far we have only compared gene expression measured at day 7 with that observed in the initial resting B cells. To characterize more fully the transcriptional differences which follow EBV or CD40L/IL4 stimulation, we next directly compared the transcriptomes of the two types of blast at day 7. In this way, we identified 255 genes which were differently expressed between EBV blasts and CD40L/IL-4 blasts (p<0.01, FC>2), of which 138 were expressed higher in EBV blasts than in CD40L/IL-4 blasts, and 117 were expressed lower in EBV blasts than in CD40L/IL-4 blasts (Data-File S2). Independent quantitative RT-qPCR assays for 40 of these differentially expressed genes confirmed >2 FC in every case (Table S1).

### Ontological profiling of the transcriptomes of EBV- and mitogen-induced B blasts

Not surprisingly, given the extent of the overlap between these datasets, the ontological profiles of genes transcriptionally changed by EBV and CD40L/IL-4 were very similar (Data-File S1), with both transcriptomes being enriched for ontological functions linked to DNA replication, cell cycle, metabolic pathways, and immune responses.

The set of genes differentially expressed between EBV blasts and CD40L/IL-4 blasts was enriched for gene functions linked to the immune response, most notably the interferon responses and cytokine/chemokine signaling pathways (Table 2). These initial analyses suggest that EBV and CD40L/IL-4 both concordantly and discordantly regulate different sets of immune response genes. To investigate these associations further, our analyses were extended to include comparisons with published array datasets in which interferon (IFN) and other cytokine responses were specifically examined.

**Table 2:** Ontology analysis of genes differentially expressed between EBV blasts and CD40L/IL-4 blasts.

Gene-sets	Ontological term	Hit count	Fold Enrichment	P Value
**A.** EBV≠CD40L/IL-4	Interferon-mediated immunity	11	11.6	<0.0001
	Cytokine/chemokine mediated immunity	9	5.2	0.0004
	B-cell- and antibody-mediated immunity	7	5.1	0.0025
	Macrophage-mediated immunity	10	4.9	0.0002
	Cytokine and chemokine signaling pathway	14	3.9	<0.0001
	T-cell mediated immunity	10	3.7	0.0016
	Immunity and defense	53	2.8	<0.0001
	Ligand-mediated signaling	14	2.4	0.0063
**B.** EBV>CD40L/IL-4	Interferon-mediated immunity	11	23.3	<0.0001
	Macrophage-mediated immunity	7	7.0	0.0005
	Immunity and defense	28	3.0	<0.0001
**C.** EBV<CD40L/IL-4	T-cell mediated immunity	9	6.5	<0.0001
	Cytokine and chemokine signaling pathway	8	4.4	0.0021
	Immunity and defense	25	2.6	<0.0001
	Cell communication	18	2.1	0.0052
	Signal transduction	39	1.6	0.0021

Ontology analysis of genes differentially expressed between EBV and CD40L/IL-4 blasts by paired LIMMA (p<0.01, FC>2) was performed using the DAVID v6.7 bioinformatics resource [28], [29] to identify Panther Biological Processes terms for which there was a significant enrichment of genes. The lists of probe-sets entered into DAVID v6.7 and the complete analyses readouts are given in Data-File S1. The probe-sets analyzed were those which were: (A) differentially expressed between EBV and CD40L/IL-4 blasts at day 7, (B) the subset of differentially expressed genes that was expressed higher in EBV blasts than in CD40L/IL-4 blasts, and (C) the subset of differentially expressed genes that was expressed lower in EBV blasts than in CD40L/IL-4 blasts.

### Interferon responses to EBV infection or CD40L/IL-4 stimulation

We first compared the transcriptional profiles of EBV and CD40L/IL-4 stimulated blasts with a list of type-I IFN-stimulated genes (ISGs) provided by Schoggins *et al* [31]; these authors had identified from a meta-analysis of published arrays 389 ISGs, of which 370 were also listed on the HuExon ST 1.0 array platform used in our experiments. Confirming the impression left by ontological profiling, this more detailed comparison (Table 3A) showed that genes up-regulated by EBV were significantly enriched for ISG (OR = 8.5, χ^2^_1df_ = 250, p<0.0001) as were those up-regulated by CD40L/IL-4, albeit to a lesser extent (OR = 3.0, χ^2^_1df_ = 25.8, p<0.0001). We also confirmed that the genes differentially expressed between EBV blasts and CD40L/IL-4 blasts at day 7 were substantially and significantly enriched for ISGs (Table 3A); this association was strongest for those genes expressed higher in EBV blasts than in CD40L/IL-4 blasts (OR = 16.8, χ^2^_1df_ = 339, p<0.0001).

**Table 3:** Expression of Interferon-regulated genes by EBV and CD40L/IL4 blasts.

Comparisons between gene sets*			overlap between gene-sets	Odds Ratio	Chi-Square	P Value (1 df)
**A.**	ISGs (n = 370) and:	Up-regulated by EBV (n = 370)	54	8.5	250	<0.0001
		Up- regulated by CD40L/IL4 (n = 370)	23	3.0	25.8	<0.0001
		Down-regulated by EBV (n = 370)	14	1.7	3.85	0.0497
		Down- regulated by CD40L/IL4 (n = 263)	15	2.7	13.9	0.0002
		Differentially expressed between EBV and CD40L/IL4 blasts (n = 249)	41	9.5	223	<0.0001
		Higher in EBV blasts than CD40L/IL4 blasts (n = 134)	35	16.8	339	<0.0001
		Lower in EBV blasts than CD40L/IL4 blasts (n = 115)	6	2.4	4.48	0.0343
**B.**	IRF-1 regulated genes (n = 113) and:	Up-regulated by EBV (n = 370)	38	24.3	494	<0.0001
		Up- regulated by CD40L/IL4 (n = 370)	16	7.4	71.1	<0.0001
		Down-regulated by EBV (n = 370)	0	-	-	-
		Down- regulated by CD40L/IL4 (n = 263)	0	-	-	-
		Differentially expressed between EBV and CD40L/IL4 blasts (n = 249)	27	22.7	373	<0.0001
		Higher in EBV blasts than CD40L/IL4 blasts (n = 134)	26	44.8	682	<0.0001
		Lower in EBV blasts than CD40L/IL4 blasts (n = 115)	1	1.3	0.055	0.815

* Numbers in parentheses indicate the number of genes within the gene-set analyzed.

Comparisons were restricted to the 16420 genes present on both the HuExon 1.0 ST and the U133 plus2 Affymetrix array platforms. The size of each comparator gene-set was selected to ensure as far as was practical that similar-sized sets were being analyzed. For example, genes up-regulated by EBV were ranked in order of FC, and the top 370 were selected for comparison with the 370 ISGs. Complete gene-lists are available in Data-File S2. (**A**) Of the 389 ISGs described by Schoggins *et al* [31] through meta-analysis of published arrays, 370 were present in the 16420 annotated genes common to both array platforms. (**B**) Of the 124 unique genes identified experimentally by Schoggins *et al* [31] as being up-regulated by IRF-1, 113 were present in the 16420 annotated genes common to both array platforms.

Similar but more substantial correlations were obtained when comparing the transcriptional profiles of EBV and CD40L/IL-4 stimulated blasts with a set of IRF-1 regulated genes identified experimentally by Schoggins *et al* [31] (Table 3B). Genes up-regulated by EBV compared with resting B cells were substantially and significantly enriched for IRF-1 regulated genes (OR = 24.3, χ^2^_1df_ = 494, p<0.0001), as were those expressed more highly in EBV blasts than in CD40L/IL-4 blasts (OR = 44.8, χ^2^_1df_ = 682, p<0.0001).

Whilst IRF-1 is an important mediator of type-I IFN responses, expression of IRF-1 transcripts themselves was only slightly and non-significantly increased at 7 days after EBV infection (FC = 1.3; p = 0.106) and non-significantly decreased in response to CD40L/IL-4 (FC = 1.4; p = 0.141).

### IL-4 signaling pathways and EBV-induced interferon responses

Given that IFNs and cytokines such as IL-4 cross-regulate each other's signaling in different cell types, including B cells [32]–[35], our analyses were extended to include comparisons with a series of array experiments by Elo *et al* [35] examining the effect of IL-4 on IL-2 stimulated CD4 T cells.

**Figure 2:**
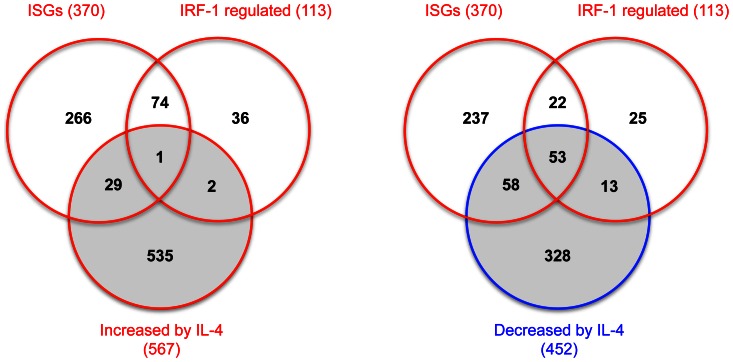
Relationships between genes regulated by Interferon, IRF-1, and IL-4. Venn diagrams showing the overlaps of 370 ISGs and 113 IRF-1 up-regulated genes defined by Schoggins et al. [31] with IL-4-regulated genes (567 up-regulated and 452 down-regulated) identified by Elo *et al* [35]. Only genes that were annotated and common to both U133 plus 2 and Human Exon 1.0 ST Affymetrix array platforms (i.e. 16420 genes) were analysed here. Complete gene-lists are available in Data-File S2.

We first confirmed a relationship between IFN and IL-4 pathways using the datasets generated by Schoggins *et al* [31] and Elo *et al* [35]. IL-4 transcriptional targets were enriched for both ISGs and IRF-1 regulated genes (Figure 2, Table 4). Most notably, as shown in Table 4B, genes that were down-regulated by IL-4 were substantially and significantly enriched both for ISGs (OR = 19.6, χ^2^_1df_ = 998, p<0.0001) and for IRF-1 induced genes (OR = 57.7, χ^2^_1df_ = 1271, p<0.0001).

**Table 4:** Expression of IL4-regulated genes by EBV-infected B blasts.

Comparisons between gene sets*			overlap between gene-sets	Odds Ratio	Chi-Square	P Value (1 df)
**A.**	Up-regulated by IL4 (n = 567) and:	ISGs (n = 370)	30	2.5	23.2	<0.0001
		IRF-1 up-regulated genes (n = 113)	3	0.76	0.209	0.648
		Up-regulated by EBV (n = 370)	12	0.94	0.047	0.828
		Up- regulated by CD40L/IL4 (n = 370)	42	3.8	66.8	<0.0001
		Down-regulated by EBV (n = 370)	35	3.0	38.7	<0.0001
		Down- regulated by CD40L/IL4 (n = 263)	17	2.0	6.90	0.009
		Differentially expressed between EBV and CD40L/IL4 blasts (n = 249)	34	4.6	75	<0.0001
		Higher in EBV blasts than CD40L/IL4 blasts (n = 134)	7	1.5	1.22	0.269
		Lower in EBV blasts than CD40L/IL4 blasts (n = 115)	27	8.9	134	<0.0001
**B.**	Down-regulated by IL4 (n = 452) and:	ISGs (n = 370)	111	19.6	998	<0.0001
		IRF-1 up-regulated genes (n = 113)	66	57.7	1271	<0.0001
		Up-regulated by EBV (n = 370)	60	7.7	244	<0.0001
		Up- regulated by CD40L/IL4 (n = 370)	27	2.9	27.8	<0.0001
		Down-regulated by EBV (n = 370)	26	2.8	24.6	<0.0001
		Down- regulated by CD40L/IL4 (n = 263)	29	4.6	65.4	<0.0001
		Differentially expressed between EBV and CD40L/IL4 blasts (n = 249)	49	9.6	259	<0.0001
		Higher in EBV blasts than CD40L/IL4 blasts (n = 134)	44	19.0	441	<0.0001
		Lower in EBV blasts than CD40L/IL4 blasts (n = 115)	5	1.6	1.06	0.303

* Numbers in parentheses indicate the number of genes within the gene-set analyzed. Comparisons were restricted to the 16420 genes present on both the HuExon 1.0 ST and the U133 plus2 Affymetrix array platforms. The IL-4 regulated genes were identified experimentally by Elo *et al* who arrayed IL-2 stimulated T cells treated with or without IL-4 [35]. Complete gene-lists are available in Data-File S2.

In light of this, and considering the relationship between subsets of EBV-regulated genes and ISGs and IRF-1 targets (Table 3), it was perhaps unsurprising that genes up-regulated by EBV were enriched for genes down-regulated by IL-4 (OR = 7.7, χ^2^_1df_ = 244, p<0.0001; Table 4B). In addition, genes that were expressed at significantly greater levels in EBV blasts than in CD40L/IL-4 blasts showed an even greater enrichment of genes down-regulated by IL-4 (OR = 19.0, χ^2^_1df_ = 441, p<0.0001; Table 4B).

**Figure 3:**
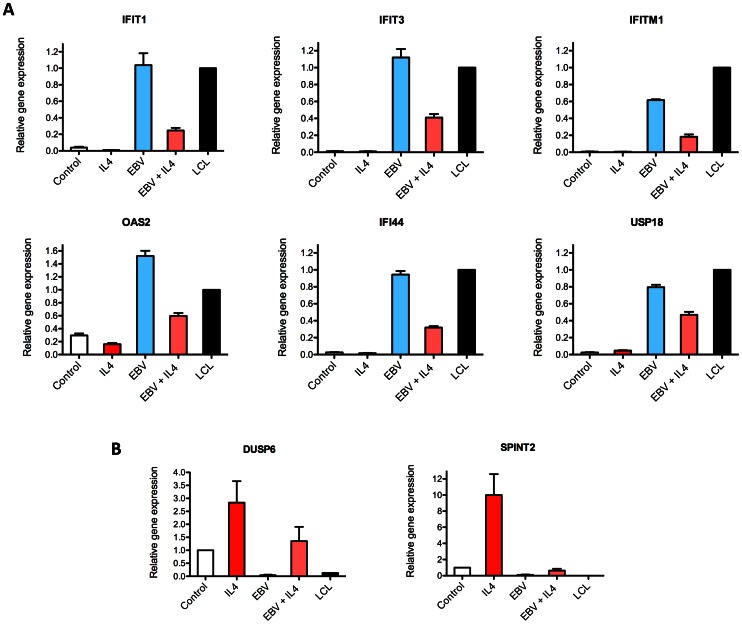
Effect of IL4 on transcription in B cells. Quantitative RT-qPCR assays for selected cellular gene transcripts in peripheral blood B cell cultures in normal medium or containing IL-4 (assayed at 6 hrs), or infected with EBV in normal medium or containing IL-4 (assayed at 7 days). The results are expressed as mean ± SEM of three replicate experiments using B cells from different donors. An established LCL was used as a reference for all assays, and all values are shown relative to LCL transcript expression = 1, except for SPINT2 and DUSP6 whose expression in the reference LCL was extremely low (ddCt<12) and where expression is shown relative to control B cell cultures. The six cellular genes shown in (**A**) were reported by Elo *et al* [35] to be down-regulated by IL4 in T cells, whilst the 2 genes shown in (**B**) were reported to be up-regulated by IL-4 in T cells.

This link between IL-4 signaling and EBV infection was confirmed directly in our B cell model by analysis of a selection of the genes reported by Elo *et al* to be down-regulated in T cells but which we found to be up-regulated following EBV infection. The results for six such genes (IFIT1, IFIT3, IFITM1, OAS2, IFI44 and USP18) are shown in Figure 3A. In each example, EBV infection caused a substantial up-regulation of transcripts but the addition of IL-4 coincident with EBV infection led to a significant down-regulation of transcripts, albeit never to the low levels detected in control uninfected cells with or without IL-4. Furthermore, when examining 2 sample genes (SPINT2, DUSP6) shown by Elo *et al* to be up-regulated by IL-4 in T cells but, in our B cell experiments to be down-regulated by EBV infection, we again found that coincident IL-4 stimulation and EBV infection attenuated the effects of EBV infection (Figure 3B).

### IL-4 impairs EBV-induced B cell transformation

**Figure 4:**
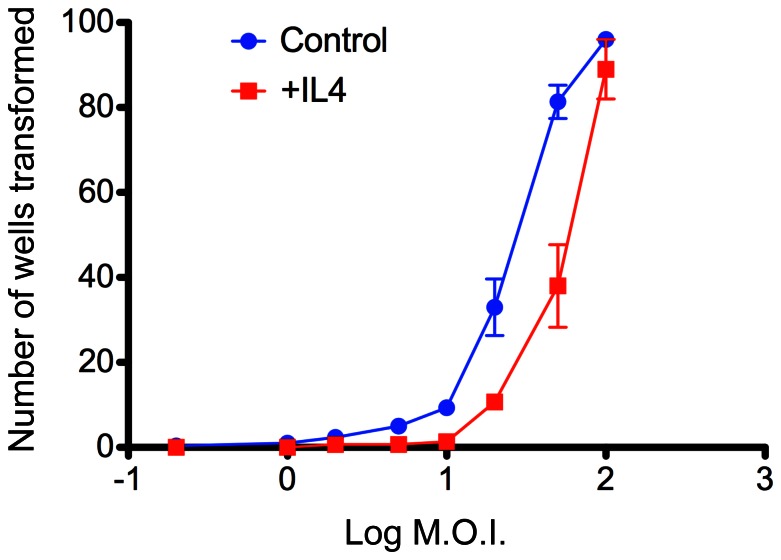
Effect of IL4 on EBV-induced B cell transformation. Purified B cells were infected with EBV at a series of multiplicities of infection (m.o.i.) in the absence or presence of added IL-4. For each infection dose, 10^3^ B cells were seeded into each of the 96-wells of a microtest plate. The cultures were maintained in the presence or absence of IL-4 for 6 weeks, at which point the number of wells containing viable transformed colonies was counted. The data points and error bars represent the mean ± s.d for replicate experiments performed on B cells from three separate donors. Transformation efficiency, defined as the M.O.I. at which 50% of the cultures were transformed, was 23.8±3.2 for EBV alone and 56.5±10.2 for EBV plus IL-4.

These analyses suggested a link between EBV-induced IFN responses and discordant regulation of IL-4 pathway genes. To elucidate the functional significance of this observation, we examined what effect IL-4 itself might have on the ability of EBV to growth-transform resting B cells. Standard growth-transformation assays were therefore set up in which purified B cells infected in the absence or presence of IL-4 over a range of EBV doses. Replicate experiments with B cells from three separate donors (Figure 4) revealed the dose of virus required for 50% transforming wells to be 23.8±3.2 (mean m.o.i. ±S.D.) for control EBV infections, and 56.5 ±10.2 for EBV plus IL-4. This represents a 2.4-fold reduction of transforming efficiency by EBV in the presence of IL-4 (p = 0.0076; paired t-test).

### Discordant regulation of IL-4 pathway genes and malignant transformation by EBV

We and others have previously shown that the transcriptomes of EBV-transformed LCL and the malignant Hodgkin and Reed-Sternberg cells (HRS) cells of HL show remarkable similarities [36], [37]. We therefore asked whether the discordant regulation of genes intersecting the IFN and IL-4 response pathways in EBV-infected B cells might also be relevant to malignant transformation in HL. As the normal counterparts of the malignant HRS cells of HL are germinal center (GC) B cells, we first determined whether the EBV/ISG/IL-4 associations could also be demonstrated in a GC B cell model of EBV infection *in vitro*. To this end, we reprised our comparisons on a previous array experiment [37] in which we had profiled germinal center (GC) B cells six weeks following their infection with EBV to yield lymphoblastoid cell lines (GC-LCLs). Consistent with our comparison of EBV and CD40L/IL-4 stimulated blasts at day 7, we found that genes up-regulated by EBV in GC-LCLs were significantly and substantially enriched for those down-regulated by IL-4 in T cells (OR = 12.0, χ^2^_1 df_ = 586, 1df p<0.0001; Table 5).

**Table 5:** Expression of IL-4 regulated genes in GC-B cell LCL.

Comparisons between gene sets*			overlap between gene-sets	Odds Ratio	Chi-Square	P Value (1 df)
**A.**	Down-regulated by IL-4 (n = 484) and:	Up-regulated by EBV (n = 484)	92	12.0	586	<0.0001
		Down- regulated by EBV (n = 484)	42	4.3	85.4	<0.0001
**B.**	Up-regulated by IL-4 (n = 640) and:	Up-regulated by EBV (n = 640)	51	2.9	50.9	<0.0001
		Down- regulated by EBV (n = 640)	52	3.0	54.2	<0.0001

* Numbers in parentheses indicate the number of genes within the gene-set analyzed.

Of the 21014 annotated genes in the U133 plus2 Affymetrix array platform, 484 were identified experimentally by Elo *et al* [35] as being down-regulated and 640 up-regulated by IL-4 in T cells. Gene-sets up- or down-regulated in EBV-transformed LCL from GC-B cells relative to uninfected GC-B cells were identified by Leonard *et al* [37]. For the determination of OR and statistical significance, equal sized probe-sets were compared by ranking probe-sets according to the magnitude of FC of the GCB-LCL relative to uninfected GC-B cells, and selecting the top 484 or 640 unique genes. Complete gene-lists are available in Data-File S2.

The transcriptional profile induced by EBV in the GC-LCL model, which we have just described, was shown previously to overlap that of HRS cells micro-dissected from biopsies of HL [37], [38]. Therefore, we next examined the overlap between IL-4 transcriptional targets and genes found to be differentially expressed in micro-dissected HRS cells from 12 cHL when compared with non-malignant GC centrocytes isolated from tonsillar lymphocytes by fluorescence activated cell sorting. We found that genes up-regulated in micro-dissected HRS cells were enriched for genes which were down-regulated by IL-4 in T cells (OR = 13.8, χ^2^_1 df_ = 724, 1df p<0.0001; Table 6).

**Table 6:** Expression of IL-4 regulated genes in HRS malignant cells.

Comparisons between gene sets*			overlap between gene-sets	Odds Ratio	Chi-Square	P Value (1 df)
**A.**	Down-regulated by IL-4 (n = 484) and:	Up-regulated in HRS (n = 484)	101	13.8	724	<0.0001
		Down- regulated in HRS (n = 484)	19	1.8	5.53	0.019
**B.**	Up-regulated by IL-4 (n = 640) and:	Up-regulated in HRS (n = 640)	56	3.2	68.4	<0.0001
		Down- regulated in HRS (n = 640)	57	3.0	59.5	<0.0001

* Numbers in parentheses indicate the number of genes within the gene-set analyzed.

IL-4-regulated genes were identified experimentally by Elo *et al* [35]; of the 21014 annotated genes in the U133 plus2 Affymetrix array platform, 640 were identified as being up-regulated and 484 down-regulated by IL4 in T cells. Genes that were up- or down-regulated in microdissected HRS malignant cells from HL biopsies relative to control GC-centrocytes were identified from the data of Brune *et al* [38]. Complete gene-lists are available in Data-File S2. For the determination of OR and statistical significance, equal sized gene-sets were compared by ranking probe-sets according to the magnitude of FC of the HRS cells, and selecting the top 640 or 484 unique genes.

**Figure 5:**
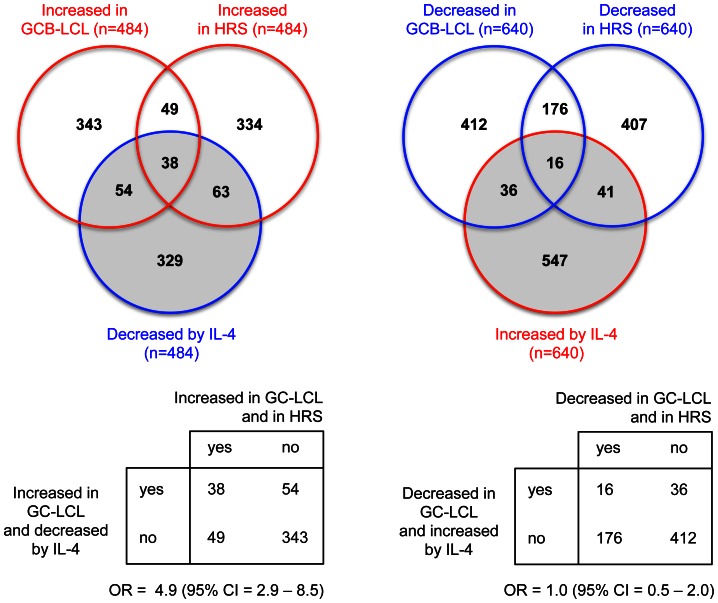
Discordant expression of IL-4 regulated transcripts by EBV and in malignant HRS cells. Gene-sets regulated by IL-4, in EBV-infected GC B cells, and in micro-dissected HRS cells were identified as described in Tables 5 and 6. To facilitate analysis of statistical significance, equal sized gene-sets were compared by ranking probe-sets according to the magnitude of FC of the HRS cells, and selecting the top 640 or 484 unique genes. Complete gene-lists are available in Data-File S2.

We hypothesized that if those EBV-induced ISGs which intersected the IL-4 signaling response and were discordantly regulated by EBV and IL-4, were to contribute to the process of transformation in HL, then genes that were up-regulated in micro-dissected HRS cells would be enriched for genes which were also up-regulated in EBV infected GC B cells but down-regulated in IL-4 treated CD4 T cells. Consistent with this hypothesis, we found that the odds of a gene up-regulated in GC-LCL cells also being up-regulated in HRS cells were almost 5 times greater (OR = 4.9; 95% CI = 2.9 to 8.5) when that gene was also down-regulated by IL-4 in T cells compared to when it was not (Figure 5). By way of a sensitivity analysis, we repeated the above analysis but this time using the transcriptional profiles generated by a recently published comparison of EBV-positive and EBV-negative micro-dissected HRS cells with micro-dissected GC cells from reactive lymph node sections [39]. Although attenuated, the point estimate of association remained significant and the 95% confidence intervals around the estimates generated by these comparisons all overlapped (Figures S1 and S2). Notably, comparison of EBV-positive and EBV-negative HRS tumors with normal GC cells gave essentially similar results (OR = 1.9, 95% CI = 1.1–3.4 for EBV-positive HRS; OR = 2.4, 95% CI = 1.4–4.3 for EBV-negative HRS).

Interestingly, no such correlation was observed when analyzing the transcriptomes of EBV-positive African endemic Burkitt lymphoma (eBL) samples arrayed by Piccaluga *et al* [40]; as shown in Figure S3, the odds of a gene up-regulated in GC-LCL cells also being up-regulated in eBL tumors were the same whether or not that gene was also down-regulated by IL-4 in T cells (OR = 1.0; 95% CI = 0.5 to 1.9).

EBV-positive cases of HL show a Latency II pattern of gene expression, in which LMP1 and LMP2 are expressed in addition to the EBNA1, EBERS, and viral miRNAs that are expressed in eBL tumors showing a Latency I pattern of viral gene expression. We therefore anticipated that those genes which were up-regulated both in HRS relative to GC-centrocytes and in EBV-transformed LCL relative to uninfected GC-B cells but down-regulated by IL-4 in T cells, might be enriched for genes known to be transcriptional targets of LMP1 or LMP2A. We identified a total of 92 signature genes fulfilling these HRS/EBV/IL-4 criteria (Table S2), and comparison with our previously published array datasets [41], [42] showed that 29/92 (32%) of these genes were indeed up-regulated by LMP1 (26 genes) or by LMP2A (5 genes) or by both (2 genes) in GC-B cells (Table 7).

**Table 7:** Regulation by LMP1 and LMP2A of EBV/HRS/IL-4 signature genes.

Category	Gene Symbol	Ontology
Genes up-regulated by LMP1 :	ATF3	transcription
	CCR7	motility, signaling
	CD274	signaling
	CD97	signaling, adhesion
	CFLAR	apoptosis
	DUSP4	signaling
	ETV6	transcription,
	IL6R	motility, apoptosis, signaling
	IRF2BP2	transcription
	LACTB	-
	LAMP3	proliferation
	LITAF	apoptosis, transcription
	MAFF	transcription, differentiation
	MIR155HG	miRNA
	MYC	proliferation, apoptosis
	PAM	protein-modification
	PERP	apoptosis
	RHEBL1	signaling
	SLAMF1	proliferation, apoptosis
	SNX9	intracellular trafficking
	SOCS2	apoptosis, signaling
	SRGN	motility, apoptosis
	TRAF1	apoptosis, signaling
	ZBTB32	transcription, DNA-repair
Genes up-regulated by both LMP1 and LMP2A :	BHLHE40	transcription
	SLAMF7	adhesion, apoptosis
Genes up-regulated by LMP2A :	CCL3	motility, signaling
	CCL4	motility, adhesion, signaling
	GNPDA1	catabolic

The EBV/HRS/IL-4 signature was defined as the 92 genes that were identified as common to the 2084 genes up-regulated in EBV-transformed LCLs established from GC-B cells [37], the 1747 genes up-regulated in HRS [38], and the 484 genes down-regulated by IL4 in T cells [35]. These 92 genes were then compared to those identified as up-regulated following expression of LMP1 or LMP2A in GC-B cells [41], [42]. All arrays were performed on the U133 plus2 Affymetrix array platform, where the probe-sets corresponded to 21014 annotated genes. The Gene Ontology function descriptions were obtained primarily from NCBI and AmiGO databases, assisted by manual literature search. Complete gene-lists are available in Data-File S2.

## Discussion

This study on the transcriptional programs of EBV infected B cells and CD40L/IL-4 stimulated B cells has identified a critical set of genes at the intersection of the IFN and IL-4 signaling pathways that modulate the transforming functions of EBV. Although the transcriptomes of EBV and CD40L/IL-4 blasts showed substantial overlap, reflecting common proliferation and apoptosis related pathways, the expression of 255 genes was found to differ significantly between the two types of blasts (p<0.01; FC>2). Initial gene ontology analysis indicated that this set of 255 genes was substantially enriched for genes involved in innate cellular responses to viral infection, most notably ISGs. At first glance, this enrichment might seem an entirely predictable consequence of the innate responses to a viral infection, which results in activation of IFN pathways aimed at preventing viral genome replication, inhibiting cell proliferation and/or inducing cell death. However, EBV infected cells are not growth-inhibited or dying any more so than CD40L/IL-4 stimulated cells.

Upon infection of cells by viruses, the ISGs are regulated in response to ligation of pattern recognition receptors (PRRs), including Toll-like receptor family members recognising virion glycoproteins or DNA/RNA [43]. EBV has the potential to activate several PRRs, including TLR3 and TLR7 [44], [45], and a role for viral RNAs, most notably the EBERs, in triggering IFN production and IFN responses is well-established [46], [47]. As with other viruses, EBV has to modulate these innate intracellular responses in order to successfully infect and persist in the cells. The prevailing evidence suggests that EBV not only evades the intended consequences of innate intracellular responses, but actually subverts them to facilitate growth-transformation [44], [48], [49]. Consistent with this property of EBV, co-stimulation of TLR9 with the CpG synthetic ligand during EBV infection of B cells *in vitro*, actually enhances LCL outgrowth [48], [49]. There are likely to be several molecular mechanisms by which EBV utilizes the pro-proliferative effects of TLR and IFN signaling whilst avoiding the anti-viral end-points of growth-inhibition and apoptosis. One intriguing example identified by Martin et al [44] was the induction of alternatively-spliced IRF-5 transcripts that inhibit the anti-proliferative effects of the regular IRF-5 isoform. Another novel possibility is suggested from our analysis of the ISGs that are differentially expressed between EBV blasts and CD40L/IL4 blasts (Table 3). Although only 6 ISGs were expressed at significantly lower levels in EBV blasts, one of these was RNF19B (expressed 2.9x lower in EBV blasts; p = 0.0012), a E3 ubiquitin-protein ligase that targets substrates such as UCKL1 for degradation; UCKL1 is a uridine kinase/uracil phosphoribosyltransferase that binds to EBV EBNA3A, an interaction that leads to a nuclear accumulation of UCKL1 that has been hypothesized to be important for EBV-induced proliferation [50].

The IFN signaling pathway is also a component of the DNA damage response (DDR) that is triggered following EBV-induced proliferation of infected B cells [51], [52]. However, it is unlikely that the DDR is responsible for the predominance of ISGs amongst those genes differentially expressed between EBV and CD40L/IL-4 blasts. Indeed, whilst we confirmed the observation of Nikitin *et al* who identified 20 DDR genes that were transcriptionally up-regulated following EBV infection [52], we also found that only one of these genes (POLQ) was expressed in CD40L/IL-4 blasts at a significantly lower level (2.7-fold; p = 0.009) than in EBV blasts.

It should be noted that whilst the genes differentially expressed by EBV and CD40L/IL-4 blasts were enriched for ISGs, a larger proportion of ISGs (58/370) was commonly regulated by both EBV and CD40L/IL-4. This begs the question, as to why some ISGs are differentially regulated between the two types of blasts and others concordantly regulated. Strikingly, we found that genes that were expressed at significantly greater levels in EBV blasts than in CD40L/IL-4 blasts were substantially and significantly enriched for genes that were down-regulated by IL-4 in other cell systems. This finding is consistent with the observation that IFNs and cytokines such as IL-4 are known to cross-regulate each other's signaling in different cell types, including B cells [32]–[35]. We were able to confirm in many cases that the induction by EBV of those ISGs that were differentially expressed in EBV blasts was reduced following infection in the presence of IL-4 (Figure 3). Furthermore, this effect of IL-4 was correlated with a significant impairment of transformation efficiency (Figure 4).

The results of these experiments are also consistent with the observation that while IL-4 can act as a potent co-stimulus for proliferation survival and differentiation in both B and T lymphocytes, hence its widespread experimental use in generating B cell blasts, it also has a modest inhibitory effect on proliferation and induces apoptosis most notably in human B cell neoplasms, and in some non-hematopoietic malignant cells [3]–[10]. They also suggest that where EBV and IL4 impact on common signaling pathways, for example, the induction of this subset of ISG, the EBV transcriptional program must prevail for transformation to occur.

It should be added that the importance of IL-4 response genes may not be confined to the ISGs that are induced by EBV; the smaller set of genes that are down-regulated by EBV but maintained or up-regulated by IL-4 includes two, SPINT2 [53], [54] and DUSP6 [55]–[57], that have been identified as candidate tumor-suppressor genes in non-hematopoietic cancers. It should be emphasized that the definition of a tumor suppressor gene is dependent upon cell context, and that whilst DUSP6 has been widely reported as having tumor suppressor function, there are reports in other cell models describing DUSP6 as an oncogene [58], [59]. Although we are unaware of any published evidence implicating DUSP6 or SPINT2 as tumor suppressors in B lymphoid malignancies, their expression was found to be increased in our experiments where IL-4 impaired EBV-induced transformation (Figures 3B, 4).

The observation that many of the transformation-associated ISGs which we have identified are overexpressed in the malignant HRS cells of HL (Figure 5, Table 6), and that a substantial number of these are also concordantly up-regulated by LMP1 or LMP2A in normal GC B cells (Table 7), suggests this maybe a profitable line of inquiry. Although IL-4 is not usually expressed in HL [60]–[62], IL13 with which it shares functional receptors [63], [64] and remarkably similar biological functions is expressed [65]. Furthermore, proliferation of HL-derived lines *in vitro* and *in vivo* is reduced by antibodies to IL-13 or by decoy receptor molecules, suggesting that IL-13 can act as an autocrine growth-factor in HL [66], [67]. Activated STAT6, which is an essential mediator of many IL-4/IL-13-regulated genes [35] is usually detected in HL along with activated STAT3 [66]. However, IFN can inhibit activation of STAT6 by cytokines [68] and is also expressed by the HRS cells of HL [69]. Furthermore, IL-13 in the lesions of EBV-positive cases of HL would be expected to induce and maintain the high levels of LMP1 expression in the absence of EBNA2 in these tumors [70], which in turn would stimulate expression of ISGs [44]. Therefore, extrapolating from our *in vitro* transformation experiments (Figures 3, 4), we suggest that the pathogenesis of HL is a consequence in part of competing and conflicting influences on genes which are at the intersection of the IFN and IL-4/IL-13 pathways, and whose dysregulation is necessary for cellular transformation.

Although the inferences we draw concerning the over-representation of ISG among genes found to be up-regulated in micro-dissected HRS cells were first predicated on an array experiment in which the EBV status of the tumors contributing to the analysis are not reported [38], we might reasonably expect 40–60% of these tumors to have had EBV in their malignant cells. Furthermore, a recent study by Steidl *et al* [39], who arrayed micro-dissected HRS cells from 7 EBV-positive and 17 EBV-negative classical HL samples, indicated a remarkable similarity in their transcriptomes; only 56 probe-sets corresponding to just 46 annotated genes were differentially expressed between the EBV-positive and –negative tumors (>2FC, p<0.01; taken from the supplementary information of the Steidl paper). This in no way undermines our conclusions, rather it adds to the body of evidence that EBV infection is but one route to the development of this tumor. For example, a major signaling function of LMP1 is to constitutively activate NF-κB [71]–[73] and many EBV-negative cases of HL carry mutations of NF-κB pathway genes that effect the same result [74], [75].

We therefore propose that the regulation by EBV of genes at the intersection of IFN and IL-4 pathways that occurs early following infection of B cells has relevance to the development or maintenance of an EBV-associated malignancy, Hodgkin's lymphoma.

## Supporting Information

Data-File S1 **Probe-lists from arrays of EBV-infection and CD40L/IL-4 stimulation of primary B cells, and results of DAVID gene-ontology analysis.**
(XLSX)

Data-File S2 **Gene-lists used for the analyses reported in** **Figures 1** **,** **3** **, and** **5** **;** **Tables 1** **and** **3** **,** **4** **,** **5** **,** **6** **,** **7** **; and Figures S1, S2, S3.**
(XLSX)

Figure S1 **Discordant expression of IL-4 regulated transcripts by EBV and in malignant EBV-positive micro-dissected HRS cells.** Analysis of gene-sets was performed as in Figure 5 except that the genes regulated in HRS cells were determined from the array data of Steidl *et al* [39], comparing the genes expressed in micro-dissected HRS cells from 7 EBV-positive HL tumors with those expressed in micro-dissected GC cells from 5 normal reactive lymph-nodes. To facilitate analysis of statistical significance, equal sized probe-sets were compared by ranking probe-sets according to the magnitude of FC of the HRS cells, and selecting the top 640 or 484 unique genes. Complete gene-lists are available in Data-File S2.
(TIF)

Figure S2 **Discordant expression of IL-4 regulated transcripts by EBV and in malignant EBV-negative micro-dissected HRS cells.** Analysis of gene-sets was performed as in Figure 5 except that the genes regulated in HRS cells were determined from the array data of Steidl *et al* [39], comparing the genes expressed in micro-dissected HRS cells from 17 EBV-negative HL tumors with those expressed in micro-dissected GC cells from 5 normal reactive lymph-nodes. To facilitate analysis of statistical significance, equal sized probe-sets were compared by ranking probe-sets according to the magnitude of FC of the HRS cells, and selecting the top 640 or 484 unique genes. Complete gene-lists are available in Data-File S2.
(TIF)

Figure S3 **Discordant expression of IL-4 regulated transcripts by EBV and in malignant eBL tumors.** Analysis of gene-sets was performed as in Figure 5 except that the genes regulated in eBL tumors were determined from the array data of Piccaluga *et al* [40], comparing the genes expressed in 9 African EBV-positive eBL tumors with those expressed in 5 samples of normal GC centroblasts. To facilitate analysis of statistical significance, equal sized probe-sets were compared by ranking probe-sets according to the magnitude of FC of the HRS cells, and selecting the top 640 or 484 unique genes. Complete gene-lists are available in Data-File S2.
(TIF)

Table S1 **Validation of RT-qPCR assays on 40 genes from the set of 255 genes identified in microarrays as significantly altered between EBV blasts and CD40L/IL-4 blasts.**
(DOCX)

Table S2 **List of 92 signature genes expressed more highly in HRS cells than in normal centrocytes, and up-regulated by EBV infection of GC B cells, and also down-regulated by IL-4 in T cells.**
(DOCX)

## References

[1] Howard M, Farrar J, Hilfiker M, Johnson B, Takatsu K, et al (1982) Identification of a T cell-derived B cell growth factor distinct from interleukin 2. J Exp Med 155: 914–923. 6977612 10.1084/jem.155.3.914 PMC2186613

[2] Banchereau J, de Paoli P, Valle A, Garcia E, Rousset F (1991) Long-term human B cell lines dependent on interleukin-4 and antibody to CD40. Science 251: 70–72. 1702555 10.1126/science.1702555

[3] Pandrau D, Saeland S, Duvert V, Durand I, Manel AM, et al (1992) Interleukin 4 inhibits in vitro proliferation of leukemic and normal human B cell precursors. J Clin Invest 90: 1697–1706. 1385474 10.1172/JCI116042 PMC443226

[4] Gooch JL, Lee AV, Yee D (1998) Interleukin 4 inhibits growth and induces apoptosis in human breast cancer cells. Cancer Res 58: 4199–4205. 9751635

[5] Obiri NI, Hillman GG, Haas GP, Sud S, Puri RK (1993) Expression of high affinity interleukin-4 receptors on human renal cell carcinoma cells and inhibition of tumor cell growth in vitro by interleukin-4. J Clin Invest 91: 88–93. 8423237 10.1172/JCI116205 PMC329999

[6] Topp MS, Papadimitriou CA, Eitelbach F, Koenigsmann M, Oelmann E, et al (1995) Recombinant human interleukin 4 has antiproliferative activity on human tumor cell lines derived from epithelial and nonepithelial histologies. Cancer Res 55: 2173–2176. 7743520

[7] Luo HY, Rubio M, Biron G, Delespesse G, Sarfati M (1991) Antiproliferative effect of interleukin-4 in B chronic lymphocytic leukemia. J Immunother (1991) 10: 418–425. 1768675 10.1097/00002371-199112000-00005

[8] Taylor CW, Grogan TM, Salmon SE (1990) Effects of interleukin-4 on the in vitro growth of human lymphoid and plasma cell neoplasms. Blood 75: 1114–1118. 2306518

[9] Lundin J, Kimby E, Bergmann L, Karakas T, Mellstedt H, et al (2001) Interleukin 4 therapy for patients with chronic lymphocytic leukaemia: a phase I/II study. Br J Haematol 112: 155–160. 11167796 10.1046/j.1365-2141.2001.02525.x

[10] Maher DW, Pike BL, Boyd AW (1990) The response of human B cells to interleukin 4 is determined by their stage of activation and differentiation. Scand J Immunol 32: 631–640. 1702898 10.1111/j.1365-3083.1990.tb03205.x

[11] Wiesner M, Zentz C, Mayr C, Wimmer R, Hammerschmidt W, et al (2008) Conditional immortalization of human B cells by CD40 ligation. PLoS ONE 3: e1464. 18213373 10.1371/journal.pone.0001464 PMC2180193

[12] Henle W, Diehl V, Kohn G, Zur Hausen H, Henle G (1967) Herpes-type virus and chromosome marker in normal leukocytes after growth with irradiated Burkitt cells. Science 157: 1064–1065. 6036237 10.1126/science.157.3792.1064

[13] Pope JH, Horne MK, Scott W (1968) Transformation of foetal human keukocytes in vitro by filtrates of a human leukaemic cell line containing herpes-like virus. Int J Cancer 3: 857–866. 4894385 10.1002/ijc.2910030619

[14] Rickinson AB, Kieff E (2007) Epstein-Barr virus. In: Knipe DM, Howley PM, editors. Fields Virology, 5th Edition. Philadelphia: Lippincott, Williams & Wilkins. pp. 2655–2700.

[15] Kieff E, Rickinson AB (2007) Epstein-Barr virus and its replication. In: Knipe DM, Howley PM, editors. Fields Virology, 5th Edition. 5th ed. Philadelphia: Lippincott, Williams & Wilkins. pp. 2603–2654.

[16] Rowe M, Kelly GL, Bell AI, Rickinson AB (2009) Burkitt's lymphoma: The Rosetta Stone deciphering Epstein-Barr virus biology. Semin Cancer Biol 19: 377–388. 19619657 10.1016/j.semcancer.2009.07.004 PMC3764430

[17] Wang D, Liebowitz D, Kieff E (1985) An EBV membrane protein expressed in immortalized lymphocytes transforms established rodent cells. Cell 43: 831–840. 3000618 10.1016/0092-8674(85)90256-9

[18] Mainou BA, Everly DN Jr, Raab-Traub N (2005) Epstein-Barr virus latent membrane protein 1 CTAR1 mediates rodent and human fibroblast transformation through activation of PI3K. Oncogene 24: 6917–6924. 16007144 10.1038/sj.onc.1208846

[19] Mosialos G, Birkenbach M, Yalamanchili R, VanArsdale T, Ware C, et al (1995) The Epstein-Barr virus transforming protein LMP1 engages signaling proteins for the Tumor Necrosis Factor receptor family. Cell 80: 389–399. 7859281 10.1016/0092-8674(95)90489-1

[20] Caldwell RG, Wilson JB, Anderson SJ, Longnecker R (1998) Epstein-Barr virus LMP2A drives B cell development and survival in the absence of normal B cell receptor signals. Immunity 9: 405–411. 9768760 10.1016/s1074-7613(00)80623-8

[21] Longnecker R, Drucker B, Roberts TM, Kieff E (1991) An Epstein-Barr virus protein associated with cell growth transformation interacts with a tyrosine kinase. Journal of Virology 65: 3681–3692. 1710288 10.1128/jvi.65.7.3681-3692.1991 PMC241385

[22] Rowe M (2001) Cell transformation induced by Epstein-Barr virus: Living dangerously. Seminars in Cancer Biology 11: 403–405. 11669601 10.1006/scbi.2000.0406

[23] Delecluse HJ, Hammerschmidt W (2000) The genetic approach to the Epstein-Barr virus: from basic virology to gene therapy. Mol Pathol 53: 270–279. 11091851 10.1136/mp.53.5.270 PMC1186980

[24] Hollyoake M, Stuhler A, Farrell P, Gordon J, Sinclair A (1995) The normal cell cycle activation program is exploited during the infection of quiescent B lymphocytes by Epstein-Barr virus. Cancer Res 55: 4784–4787. 7585505

[25] Delecluse HJ, Hilsendegen T, Pich D, Zeidler R, Hammerschmidt W (1998) Propagation and recovery of intact, infectious Epstein-Barr virus from prokaryotic to human cells. Proc Natl Acad Sci USA 95: 8245–8250. 9653172 10.1073/pnas.95.14.8245 PMC20961

[26] Shannon-Lowe C, Baldwin G, Feederle R, Bell A, Rickinson A, et al (2005) Epstein-Barr virus-induced B-cell transformation: quantitating events from virus binding to cell outgrowth. J Gen Virol 86: 3009–3019. 16227222 10.1099/vir.0.81153-0

[27] Smyth GK (2004) Linear models and empirical bayes methods for assessing differential expression in microarray experiments. Stat Appl Genet Mol Biol 3: Article3. 16646809 10.2202/1544-6115.1027

[28] Huang DW, Sherman BT, Lempicki RA (2008) Systematic and integrative analysis of large gene lists using DAVID bioinformatics resources. Nat Protocols 4: 44–57. 10.1038/nprot.2008.211 19131956

[29] Huang DW, Sherman BT, Lempicki RA (2009) Bioinformatics enrichment tools: paths toward the comprehensive functional analysis of large gene lists. Nucleic Acids Res 37: 1–13. 19033363 10.1093/nar/gkn923 PMC2615629

[30] Livak KJ, Schmittgen TD (2001) Analysis of relative gene expression data using real-time quantitative PCR and the 2(−Delta Delta C(T)) Method. Methods 25: 402–408. 11846609 10.1006/meth.2001.1262

[31] Schoggins JW, Wilson SJ, Panis M, Murphy MY, Jones CT, et al (2011) A diverse range of gene products are effectors of the type I interferon antiviral response. Nature 472: 481–485. 21478870 10.1038/nature09907 PMC3409588

[32] Kim SH, Lee CE (2011) Counter-regulation mechanism of IL-4 and IFN-alpha signal transduction through cytosolic retention of the pY-STAT6:pY-STAT2:p48 complex. Eur J Immunol 41: 461–472. 21268015 10.1002/eji.201040668

[33] Larner AC, Petricoin EF, Nakagawa Y, Finbloom DS (1993) IL-4 attenuates the transcriptional activation of both IFN-alpha and IFN-gamma-induced cellular gene expression in monocytes and monocytic cell lines. J Immunol 150: 1944–1950. 8436826

[34] Minamino K, Takahara K, Adachi T, Nagaoka K, Iyoda T, et al (2012) IRF-2 regulates B-cell proliferation and antibody production through distinct mechanisms. Int Immunol 24: 573–581. 22773153 10.1093/intimm/dxs060

[35] Elo LL, Jarvenpaa H, Tuomela S, Raghav S, Ahlfors H, et al (2010) Genome-wide profiling of interleukin-4 and STAT6 transcription factor regulation of human Th2 cell programming. Immunity 32: 852–862. 20620947 10.1016/j.immuni.2010.06.011

[36] Kuppers R, Klein U, Schwering I, Distler V, Brauninger A, et al (2003) Identification of Hodgkin and Reed-Sternberg cell-specific genes by gene expression profiling. J Clin Invest 111: 529–537. 12588891 10.1172/JCI16624 PMC151925

[37] Leonard S, Wei W, Anderton J, Vockerodt M, Rowe M, et al (2011) Epigenetic and transcriptional changes which follow Epstein-Barr Virus infection of germinal center B cells and their relevance to the pathogenesis of Hodgkin's lymphoma. J Virol 85: 9568–9577. 21752916 10.1128/JVI.00468-11 PMC3165764

[38] Brune V, Tiacci E, Pfeil I, Doring C, Eckerle S, et al (2008) Origin and pathogenesis of nodular lymphocyte-predominant Hodgkin lymphoma as revealed by global gene expression analysis. J Exp Med 205: 2251–2268. 18794340 10.1084/jem.20080809 PMC2556780

[39] Steidl C, Diepstra A, Lee T, Chan FC, Farinha P, et al (2012) Gene expression profiling of microdissected Hodgkin Reed-Sternberg cells correlates with treatment outcome in classical Hodgkin lymphoma. Blood 120: 3530–3540. 22955918 10.1182/blood-2012-06-439570

[40] Piccaluga PP, De Falco G, Kustagi M, Gazzola A, Agostinelli C, et al (2011) Gene expression analysis uncovers similarity and differences among Burkitt lymphoma subtypes. Blood 117: 3596–3608. 21245480 10.1182/blood-2010-08-301556

[41] Vockerodt M, Morgan SL, Kuo M, Wei W, Chukwuma MB, et al (2008) The Epstein-Barr virus oncoprotein, latent membrane protein-1, reprograms germinal centre B cells towards a Hodgkin's Reed-Sternberg-like phenotype. J Pathol 216: 83–92. 18566961 10.1002/path.2384

[42] Vockerodt M, Wei W, Nagy E, Prouzova Z, Schrader A, et al (2013) Suppression of the LMP2a target gene, Egr-1, protects Hodgkin's lymphoma cells from entry to the EBV lytic cycle. J Pathol DOI: 10.1002/path.4198. 10.1002/path.4198 23592216

[43] Takeuchi O, Akira S (2010) Pattern recognition receptors and inflammation. Cell 140: 805–820. 20303872 10.1016/j.cell.2010.01.022

[44] Martin HJ, Lee JM, Walls D, Hayward SD (2007) Manipulation of the toll-like receptor 7 signaling pathway by Epstein-Barr virus. J Virol 81: 9748–9758. 17609264 10.1128/JVI.01122-07 PMC2045431

[45] Iwakiri D, Zhou L, Samanta M, Matsumoto M, Ebihara T, et al (2009) Epstein-Barr virus (EBV)-encoded small RNA is released from EBV-infected cells and activates signaling from Toll-like receptor 3. J Exp Med 206: 2091–2099. 19720839 10.1084/jem.20081761 PMC2757889

[46] Jochum S, Ruiss R, Moosmann A, Hammerschmidt W, Zeidler R (2012) RNAs in Epstein-Barr virions control early steps of infection. Proc Natl Acad Sci U S A 109: E1396–1404. 22543160 10.1073/pnas.1115906109 PMC3361417

[47] Samanta M, Iwakiri D, Kanda T, Imaizumi T, Takada K (2006) EB virus-encoded RNAs are recognized by RIG-I and activate signaling to induce type I IFN. EMBO J 25: 4207–4214. 16946700 10.1038/sj.emboj.7601314 PMC1570431

[48] Traggiai E, Becker S, Subbarao K, Kolesnikova L, Uematsu Y, et al (2004) An efficient method to make human monoclonal antibodies from memory B cells: potent neutralization of SARS coronavirus. Nat Med 10: 871–875. 15247913 10.1038/nm1080 PMC7095806

[49] Iskra S, Kalla M, Delecluse HJ, Hammerschmidt W, Moosmann A (2010) Toll-like receptor agonists synergistically increase proliferation and activation of B cells by epstein-barr virus. J Virol 84: 3612–3623. 20089650 10.1128/JVI.01400-09 PMC2838115

[50] Kashuba E, Kashuba V, Sandalova T, Klein G, Szekely L (2002) Epstein-Barr virus encoded nuclear protein EBNA-3 binds a novel human uridine kinase/uracil phosphoribosyltransferase. BMC Cell Biol 3: 23. 12199906 10.1186/1471-2121-3-23 PMC126255

[51] Brzostek-Racine S, Gordon C, Van Scoy S, Reich NC (2011) The DNA damage response induces IFN. J Immunol 187: 5336–5345. 22013119 10.4049/jimmunol.1100040 PMC3246365

[52] Nikitin PA, Yan CM, Forte E, Bocedi A, Tourigny JP, et al (2010) An ATM/Chk2-mediated DNA damage-responsive signaling pathway suppresses Epstein-Barr virus transformation of primary human B cells. Cell Host Microbe 8: 510–522. 21147465 10.1016/j.chom.2010.11.004 PMC3049316

[53] Morris MR, Gentle D, Abdulrahman M, Maina EN, Gupta K, et al (2005) Tumor suppressor activity and epigenetic inactivation of hepatocyte growth factor activator inhibitor type 2/SPINT2 in papillary and clear cell renal cell carcinoma. Cancer Res 65: 4598–4606. 15930277 10.1158/0008-5472.CAN-04-3371

[54] Kongkham PN, Northcott PA, Ra YS, Nakahara Y, Mainprize TG, et al (2008) An epigenetic genome-wide screen identifies SPINT2 as a novel tumor suppressor gene in pediatric medulloblastoma. Cancer Res 68: 9945–9953. 19047176 10.1158/0008-5472.CAN-08-2169

[55] Furukawa T, Fujisaki R, Yoshida Y, Kanai N, Sunamura M, et al (2005) Distinct progression pathways involving the dysfunction of DUSP6/MKP-3 in pancreatic intraepithelial neoplasia and intraductal papillary-mucinous neoplasms of the pancreas. Mod Pathol 18: 1034–1042. 15832194 10.1038/modpathol.3800383

[56] Furukawa T, Sunamura M, Motoi F, Matsuno S, Horii A (2003) Potential tumor suppressive pathway involving DUSP6/MKP-3 in pancreatic cancer. Am J Pathol 162: 1807–1815. 12759238 10.1016/S0002-9440(10)64315-5 PMC1868131

[57] Xu S, Furukawa T, Kanai N, Sunamura M, Horii A (2005) Abrogation of DUSP6 by hypermethylation in human pancreatic cancer. J Hum Genet 50: 159–167. 15824892 10.1007/s10038-005-0235-y

[58] Degl'innocenti D, Romeo P, Tarantino E, Sensi M, Cassinelli G, et al (2013) DUSP6/MKP3 is overexpressed in papillary and poorly differentiated thyroid carcinoma and contributes to neoplastic properties of thyroid cancer cells. Endocr Relat Cancer 20: 23–37. 23132790 10.1530/ERC-12-0078

[59] Messina S, Frati L, Leonetti C, Zuchegna C, Di Zazzo E, et al (2011) Dual-specificity phosphatase DUSP6 has tumor-promoting properties in human glioblastomas. Oncogene 30: 3813–3820. 21499306 10.1038/onc.2011.99

[60] Herbst H, Foss HD, Samol J, Araujo I, Klotzbach H, et al (1996) Frequent expression of interleukin-10 by Epstein-Barr virus-harboring tumor cells of Hodgkin's disease. Blood 87: 2918–2929. 8639912

[61] Merz H, Fliedner A, Orscheschek K, Binder T, Sebald W, et al (1991) Cytokine expression in T-cell lymphomas and Hodgkin's disease. Its possible implication in autocrine or paracrine production as a potential basis for neoplastic growth. Am J Pathol 139: 1173–1180. 1951632 PMC1886335

[62] Serrano D, Ghiotto F, Roncella S, Airoldi I, Cutrona G, et al (1997) The patterns of IL2, IFN-gamma, IL4 and IL5 gene expression in Hodgkin's disease and reactive lymph nodes are similar. Haematologica 82: 542–549. 9407718

[63] Junttila IS, Mizukami K, Dickensheets H, Meier-Schellersheim M, Yamane H, et al (2008) Tuning sensitivity to IL-4 and IL-13: differential expression of IL-4Ralpha, IL-13Ralpha1, and gammac regulates relative cytokine sensitivity. J Exp Med 205: 2595–2608. 18852293 10.1084/jem.20080452 PMC2571934

[64] Umeshita-Suyama R, Sugimoto R, Akaiwa M, Arima K, Yu B, et al (2000) Characterization of IL-4 and IL-13 signals dependent on the human IL-13 receptor alpha chain 1: redundancy of requirement of tyrosine residue for STAT3 activation. Int Immunol 12: 1499–1509. 11058569 10.1093/intimm/12.11.1499

[65] Zurawski G, de Vries JE (1994) Interleukin 13, an interleukin 4-like cytokine that acts on monocytes and B cells, but not on T cells. Immunol Today 15: 19–26. 7907877 10.1016/0167-5699(94)90021-3

[66] Skinnider BF, Elia AJ, Gascoyne RD, Patterson B, Trumper L, et al (2002) Signal transducer and activator of transcription 6 is frequently activated in Hodgkin and Reed-Sternberg cells of Hodgkin lymphoma. Blood 99: 618–626. 11781246 10.1182/blood.v99.2.618

[67] Trieu Y (2004) Soluble Interleukin-13R 2 Decoy Receptor Inhibits Hodgkin's Lymphoma Growth in Vitro and in Vivo. Cancer Research 64: 3271–3275. 15126369 10.1158/0008-5472.can-03-3764

[68] Dickensheets HL, Venkataraman C, Schindler U, Donnelly RP (1999) Interferons inhibit activation of STAT6 by interleukin 4 in human monocytes by inducing SOCS-1 gene expression. Proc Natl Acad Sci U S A 96: 10800–10805. 10485906 10.1073/pnas.96.19.10800 PMC17963

[69] Gerdes J, Kretschmer C, Zahn G, Ernst M, Jones DB, et al (1990) Immunoenzymatic assessment of interferon-gamma in Hodgkin and Sternberg-Reed cells. Cytokine 2: 307–310. 2129505 10.1016/1043-4666(90)90033-p

[70] Kis LL, Gerasimcik N, Salamon D, Persson EK, Nagy N, et al (2011) STAT6 signaling pathway activated by the cytokines IL-4 and IL-13 induces expression of the Epstein-Barr virus-encoded protein LMP-1 in absence of EBNA-2: implications for the type II EBV latent gene expression in Hodgkin lymphoma. Blood 117: 165–174. 20876453 10.1182/blood-2010-01-265272

[71] Laherty CD, Hu HM, Opipari AW, Wang F, Dixit VM (1992) Epstein-Barr virus LMP1 gene product induces A20 zinc finger protein expression by activating nuclear factor kB. J Biol Chem 267: 24157–24160. 1332946

[72] Hammarskjöld M-L, Simurda MC (1992) Epstein-Barr virus latent membrane protein transactivates the human immunodeficiency virus type 1 long terminal repeat through induction of NF-κB activity. Journal of Virology 66: 6496–6501. 1404600 10.1128/jvi.66.11.6496-6501.1992 PMC240142

[73] Huen DS, Henderson SH, Croom-Carter D, Rowe M (1995) The Epstein-Barr virus Latent Membrane Protein (LMP1) mediates activation of NF-kB and cell surface phenotype via two effector regions in its carboxyl-terminal cytoplasmic domain. Oncogene 10: 549–560. 7845680

[74] Cabannes E, Khan G, Aillet F, Jarrett RF, Hay RT (1999) Mutations in the IkBa gene in Hodgkin's disease suggest a tumour suppressor role for IkappaBalpha. Oncogene 18: 3063–3070. 10340377 10.1038/sj.onc.1202893

[75] Kuppers R (2009) The biology of Hodgkin's lymphoma. Nat Rev Cancer 9: 15–27. 19078975 10.1038/nrc2542

