# Supplementary figures and images for: Induction of Interferon-Stimulated Genes on the IL-4 Response Axis by Epstein-Barr Virus Infected Human B Cells; Relevance to Cellular Transformation
Source: PLoS One. 2013 May 27;8(5):e64868. doi: 10.1371/journal.pone.0064868 (PMC3664578; doi:10.1371/journal.pone.0064868)

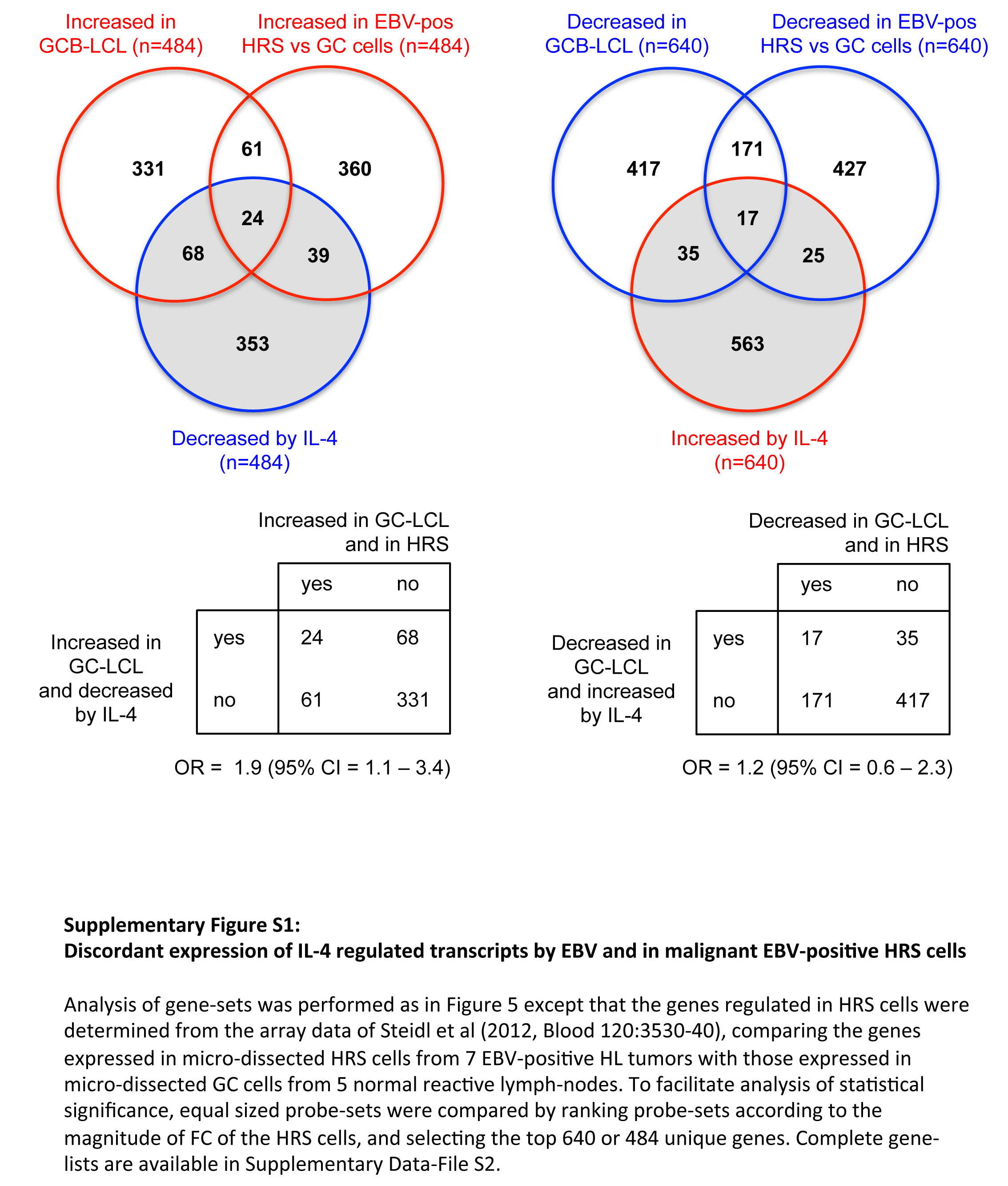

Supplement: Figure S1 — Discordant expression of IL-4 regulated transcripts by EBV and in malignant EBV-positive micro-dissected HRS cells. Analysis of gene-sets was performed as in Figure 5 except that the genes regulated in HRS cells were determined from the array data of Steidl et al [39], comparing the genes expressed in micro-dissected HRS cells from 7 EBV-positive HL tumors with those expressed in micro-dissected GC cells from 5 normal reactive lymph-nodes. To facilitate analysis of statistical significance, equal sized probe-sets were compared by ranking probe-sets according to the magnitude of FC of the HRS cells, and selecting the top 640 or 484 unique genes. Complete gene-lists are available in Data-File S2. (TIF) [file pone.0064868.s003.tif]

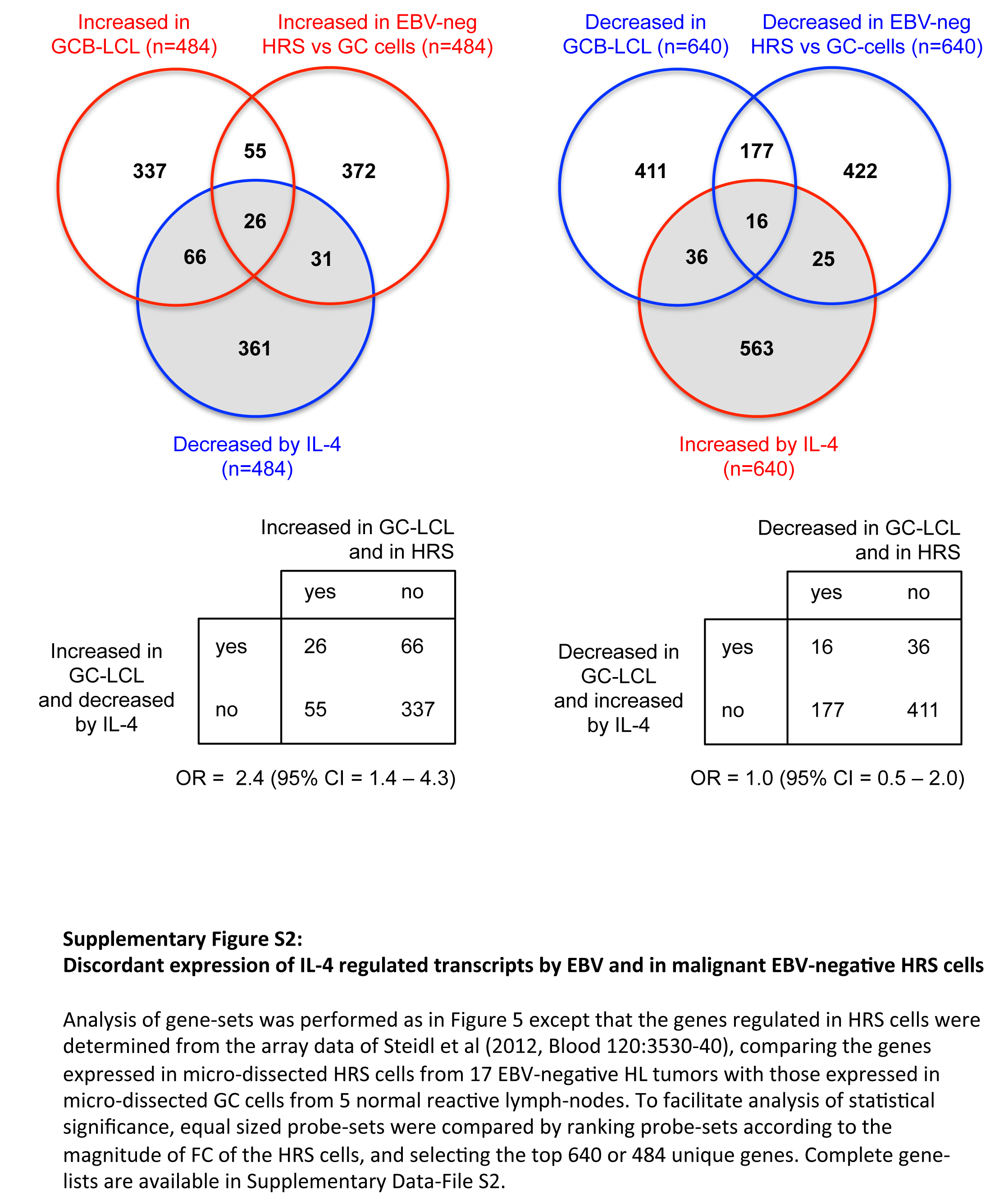

Supplement: Figure S2 — Discordant expression of IL-4 regulated transcripts by EBV and in malignant EBV-negative micro-dissected HRS cells. Analysis of gene-sets was performed as in Figure 5 except that the genes regulated in HRS cells were determined from the array data of Steidl et al [39], comparing the genes expressed in micro-dissected HRS cells from 17 EBV-negative HL tumors with those expressed in micro-dissected GC cells from 5 normal reactive lymph-nodes. To facilitate analysis of statistical significance, equal sized probe-sets were compared by ranking probe-sets according to the magnitude of FC of the HRS cells, and selecting the top 640 or 484 unique genes. Complete gene-lists are available in Data-File S2. (TIF) [file pone.0064868.s004.tif]

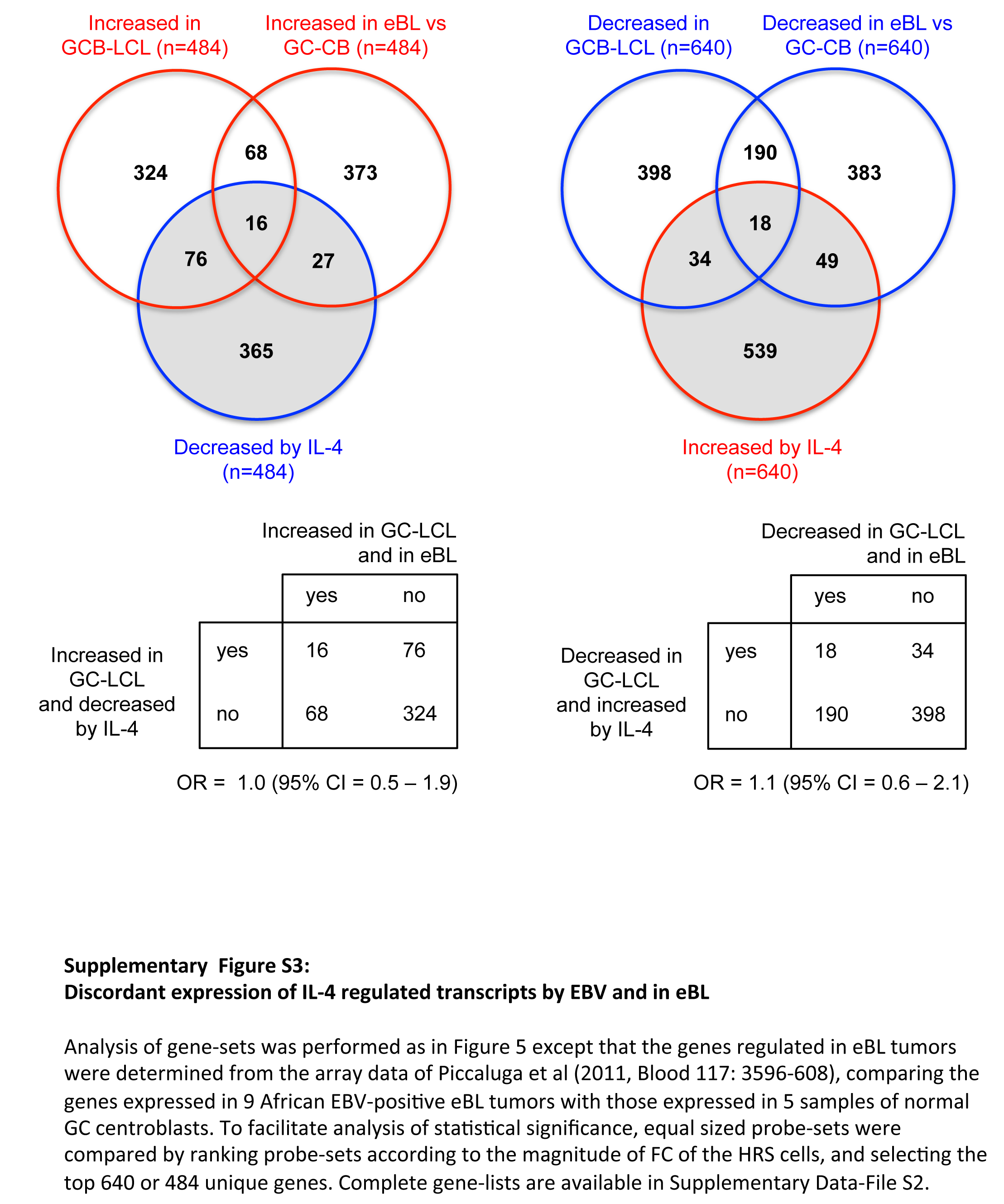

Supplement: Figure S3 — Discordant expression of IL-4 regulated transcripts by EBV and in malignant eBL tumors. Analysis of gene-sets was performed as in Figure 5 except that the genes regulated in eBL tumors were determined from the array data of Piccaluga et al [40], comparing the genes expressed in 9 African EBV-positive eBL tumors with those expressed in 5 samples of normal GC centroblasts. To facilitate analysis of statistical significance, equal sized probe-sets were compared by ranking probe-sets according to the magnitude of FC of the HRS cells, and selecting the top 640 or 484 unique genes. Complete gene-lists are available in Data-File S2. (TIF) [file pone.0064868.s005.tif]
