# Supplementary material for: Induction of Interferon-Stimulated Genes on the IL-4 Response Axis by Epstein-Barr Virus Infected Human B Cells; Relevance to Cellular Transformation
Source: PLoS One. 2013 May 27;8(5):e64868. doi: 10.1371/journal.pone.0064868 (PMC3664578; doi:10.1371/journal.pone.0064868)
Supplement: Table S1 — Validation of RT-qPCR assays on 40 genes from the set of 255 genes identified in microarrays as significantly altered between EBV blasts and CD40L/IL-4 blasts. (DOCX) [file pone.0064868.s006.docx]

**Table S1: Validation RTqPCR assays on 40 genes within the set of 255 genes identified in microarrays as significantly altered between EBV blasts and CD40L/IL-4 blasts.**

| **Assay** | **Gene** | **Fold-change in EBV blasts vs resting B cells^a^** | **Fold-change in CD40L/IL-4 blasts vs resting B cells^a^** | **Fold-change in EBV blasts vs CD40L/IL-4 blasts^a^** | **P Value^b^** |
| --- | --- | --- | --- | --- | --- |
| Hs00171144_m1 | CCL25 | +561.8 | +5.9 | +95.4 | 0.049 |
| Hs00356631_g1 | IFIT1 | +324.2 | +9.5 | +34.2 | 0.020 |
| Hs00235001_m1 | NR4A3 | –16.3 | +1.6 | –26.7 | 0.002 |
| Hs00369813_m1 | RSAD2 | +32.7 | +1.6 | +21.0 | 0.056 |
| Hs00197427_m1 | IFI44 | +11.5 | –1.8 | +20.5 | 0.061 |
| Hs00381974_m1 | CD300A | +48.0 | +2.5 | +19.1 | 0.014 |
| Hs00169257_m1 | DUSP6 | –9.1 | +1.8 | –16.3 | 0.001 |
| Hs00155486_m1 | IL12RB2 | +53.4 | +3.4 | +15.6 | 0.028 |
| Hs00983152_m1 | NETO2 | +5.4 | –2.8 | +15.2 | 0.086 |
| Hs00173936_m1 | SPINT2 | –3.6 | +3.2 | –11.5 | 0.016 |
| Hs00930963_m1 | PLAC8 | +1.8 | –5.9 | +10.9 | 0.016 |
| Hs00225412_m1 | ELOVL6 | +92.5 | +8.5 | +10.8 | 0.065 |
| Hs01068508_m1 | PRDM1 | +2.9 | –3.7 | +10.8 | 0.007 |
| UHs00276441_m1 | USP18 | +214.1 | +20.3 | +10.6 | 0.010 |
| Hs00242571_m1 | IFI6 | +14.2 | +1.4 | +10.2 | 0.012 |
| Hs00382744_m1 | IFIT3 | +44.9 | +4.6 | +9.8 | 0.078 |
| Hs00992389_m1 | MAP2K6 | +10.8 | +1.3 | +8.3 | 0.035 |
| Hs00275514_m1 | PLSCR1 | +9.6 | +1.3 | +7.5 | 0.008 |
| Hs00292828_m1 | CABLES1 | +88.5 | +12.5 | +7.1 | 0.005 |
| Hs00400624_m1 | GPR155 | +5.3 | –1.3 | +6.9 | 0.046 |
| Hs00895598_m1 | MX1 | +19.7 | +2.9 | +6.8 | 0.002 |
| Hs00187790_m1 | CHI3L2 | +13.1 | +2.1 | +6.4 | 0.020 |
| Hs00159418_m1 | MX2 | +17.6 | +2.9 | +6.1 | 0.004 |
| Hs00388714_m1 | OASL | +5.2 | –1.1 | +5.9 | 0.034 |
| Hs00196324_m1 | OAS3 | +24.2 | +4.6 | +5.3 | 0.001 |
| Hs00159719_m1 | OAS2 | +37.0 | +7.5 | +5.0 | 0.016 |
| IHs01652522_g1 | IFITM1 | +11.7 | +2.4 | +4.9 | 0.003 |
| Hs00261581_m1 | CARD6 | +77.9 | +17.0 | +4.6 | 0.072 |
| Hs01103591_m1 | PIK3R3 | +25.8 | +6.1 | +4.2 | 0.150 |
| Hs00223114_m1 | SLC44A1 | +6.5 | +1.7 | +4.0 | 0.022 |
| Hs00169491_m1 | STK3 | +5.0 | +1.4 | +3.6 | 0.074 |
| Hs00174774_m1 | LGALS3BP | +22.9 | +7.1 | +3.3 | 0.014 |
| Hs00559848_m1 | LGMN | +3.0 | –1.1 | +3.2 | 0.018 |
| Hs00158942_m1 | LY6E | +1.6 | –2.2 | +2.9 | 0.020 |
| Hs00377819_m1 | RNASE6 | +2.1 | –1.4 | +2.8 | 0.010 |
| Hs00602615_m1 | CECR1 | +1.7 | –1.4 | +2.8 | 0.015 |
| Hs00845785_m1 | PTP4A3 | +3.5 | +1.3 | +2.6 | 0.053 |
| Hs00220172_m1 | C3orf37 | +2.9 | +1.2 | +2.5 | 0.041 |
| Hs00414000_m1 | FCGR2B | +4.7 | +2.0 | +2.4 | 0.076 |
| Hs00231947_m1 | TRIM25 | +5.7 | +2.6 | +2.2 | 0.006 |

**^a^** Red shaded boxes indicate >2-fold increase, blue shaded boxes indicate >2-fold decrease.

**^b^** P value from Student paired t test, comparing paired expression values for EBV blasts and CD40L/IL-4 blasts in three replicate experiments with different donor peripheral blood B cells. All genes showed >2FC.
