# Supplementary material for: Induction of Interferon-Stimulated Genes on the IL-4 Response Axis by Epstein-Barr Virus Infected Human B Cells; Relevance to Cellular Transformation
Source: PLoS One. 2013 May 27;8(5):e64868. doi: 10.1371/journal.pone.0064868 (PMC3664578; doi:10.1371/journal.pone.0064868)
Supplement: Table S2 — List of 92 signature genes expressed more highly in HRS cells than in normal centrocytes, and up-regulated by EBV infection of GC B cells, and also down-regulated by IL-4 in T cells. (DOCX) [file pone.0064868.s007.docx]

**Table S2:**

**List of 92 signature genes expressed more highly in HRS cells than normal centrocytes, and up-regulated by EBV infection of GC B cells, and also down-regulated by IL-4 in T cells**

| ANXA2 | | | |
| --- | --- | --- | --- |
| APOL3 | | | |
| ATF3 | | | |
| BBIP1 | | | |
| BCL2L14 | | | |
| BHLHE40 | | | |
| BLVRA | | | |
| C9orf46 | | | |
| CCL3 /// CCL3L1 /// CCL3L3 | | | |
| CCL4 | | | |
| CCND2 | | | |
| CCR7 | | | |
| CD274 | | | |
| CD97 | | | |
| CDKN1A | | | |
| CFLAR | | | |
| CYFIP1 | | | |
| DDIT4 | | | |
| DTX3L | | | |
| DUSP4 | | | |
| EMR1 | | | |
| ETV6 | | | |
| FBXO6 | | | |
| FLOT1 | | | |
| GIMAP6 | | | |
| GLUL | | | |
| GNA15 | | | |
| GNPDA1 | | | |
| HAVCR2 | | | |
| HDGFRP3 | | | |
| IFI30 | | | |
| IFI35 | |  |  |
| IFI6 | |  |  |
| IFIT3 | |  |  |
| IFITM1 | |  |  |
| IFITM2 | |  |  |
| IFITM3 | |  |  |
| IL1R2 | |  |  |
| IL2RB | |  |  |
| IL6R | |  |  |
| IPCEF1 | |  |  |
| IRF2BP2 | |  |  |
| ISG15 | |  |  |
| KDSR | |  |  |
| KSR1 | |  |  |
| LACTB | |  |  |
| LAG3 | |  |  |
| LAMP3 | |  |  |
| LAP3 | |  |  |
| LGALS3 | |  |  |
| LGMN | |  |  |
| LITAF | |  |  |
| LUZP1 | |  |  |
| MAFF | |  |  |
| MB21D2 | |  |  |
| MIR155HG | |  |  |
| MSRB2 | |  |  |
| MT1X | |  |  |
| MVP | |  |  |
| MYC | |  |  |
| NOL3 | |  |  |
| OASL | |  |  |
| OPTN | |  |  |
| OXNAD1 | |  |  |
| PAM | |  |  |
| PERP | |  |  |
| PPIF | |  |  |
| PSME2 | |  |  |
| RAB13 | |  |  |
| RBCK1 | |  |  |
| RHEBL1 | |  |  |
| RORA | |  |  |
| SAMHD1 | |  |  |
| SLAMF1 | |  |  |
| SLAMF7 | |  |  |
| SNX9 | |  |  |
| SOCS2 | |  |  |
| SPATS2L | |  |  |
| SQRDL | |  |  |
| SRGN | |  |  |
| STAT1 | |  |  |
| STAT3 | |  |  |
| TAP1 | |  |  |
| TNFRSF1A | |  |  |
| TNFRSF1B | |  |  |
| TNFSF13B | |  |  |
| TNIK | |  |  |
| TRAF1 | |  |  |
| UBE2L6 | |  |  |
| WARS | |  |  |
| ZBTB32 | |  |  |
| ZNRF1 | |  |  |

The overlap between 1,747 genes up-regulated in HRS (Brune *et al,* 2008, J Exp Med 2008, 205:2251) and the 2,084 genes up-regulated in EBV-transformed LCLs established from GC-B cells (Leonard *et al,* 2011, J Virol 85:9568) and the 484 genes down regulated by IL-4 in T cells (Elo *et al*, 2010, Immunity 32:852). Comparison of these three sets of genes identified an overlap of 92 genes common to each.
